# Inherited thrombocytopenia: novel insights into megakaryocyte maturation, proplatelet formation and platelet lifespan

**DOI:** 10.3109/09537104.2016.1148806

**Published:** 2016-03-30

**Authors:** Ben Johnson, Sarah J. Fletcher, Neil V. Morgan

**Affiliations:** ^a^ Institute of Cardiovascular Sciences, College of Medical and Dental Sciences, University of Birmingham, UK

**Keywords:** Inherited thrombocytopenia, megakaryocytes, platelets, gene mutations, bleeding

## Abstract

The study of patients with inherited bleeding problems is a powerful approach in determining the function and regulation of important proteins in human platelets and their precursor, the megakaryocyte. The normal range of platelet counts in the bloodstream ranges from 150 000 to 400 000 platelets per microliter and is normally maintained within a narrow range for each individual. This requires a constant balance between thrombopoiesis, which is primarily controlled by the cytokine thrombopoietin (TPO), and platelet senescence and consumption. Thrombocytopenia can be defined as a platelet count of less than 150 000 per microliter and can be acquired or inherited. Heritable forms of thrombocytopenia are caused by mutations in genes involved in megakaryocyte differentiation, platelet production and platelet removal. In this review, we will discuss the main causative genes known for inherited thrombocytopenia and highlight their diverse functions and whether these give clues on the processes of platelet production, platelet function and platelet lifespan. Additionally, we will highlight the recent advances in novel genes identified for inherited thrombocytopenia and their suggested function.

## Introduction

Inherited thombocytopenias (ITs) are a heterogeneous group of disorders characterized by a sustained reduction in platelet count manifesting as a bleeding diathesis. Since the discovery of disease inheritance patterns in disorders such as Bernard Soulier Syndrome (BSS), genetic studies of thrombocytopenia have been a vital tool in determining megakaryocyte and platelet physiology [1]. As a result of parallel whole exome and whole genome sequencing over the past 5–10 years, we are discovering increasing numbers of novel genes with a critical role in platelet physiology. However, are any of these genes giving us clues to platelet lifespan? Is IT predominately caused by a defect in the process of platelet production or is there more to the story than we currently know?

To date, there are 31 genes suspected to cause 27 separate forms of inherited thrombocytopenia (Table I). The majority of patients with thrombocytopenia present secondary to syndromic disorders usually affecting the immune system or in cases of immune thrombocytopenic purpura (ITP). However, a number of cases present as a bleeding diathesis as a result of a reduced platelet count, sometimes alongside additional platelet dysfunction, indicating an effect solely on cells of the hematopoietic lineage. As a consequence of the varied phenotypic display and clinical presentation of inherited thrombocytopenias, there are several ways in which they are characterized. One such way, which has been favored following molecular characterization of the effect of mutations, is to group genes based upon their effect on megakaryocyte differentiation, platelet production and platelet function. Therefore, to understand whether a link to platelet lifespan can be established it is best to first consider all genes implicated in a reduced platelet count.

**Table I.** Genetic causes of inherited thrombocytopenia, the encoded protein and their associated diseases. Grouped into their suggested role within megakaryopoiesis, platelet production or clearance/other.

Area of mutational effect	Gene	Protein	Associated disease	References
Megakaryopoiesis	*ANKRD26*	Ankyrin repeat domain 26	ANKRD26-related thrombocytopenia	[25]
	*ETV6*	Transcription factor ETV6	THC5	[7]
	*FLI1*	Friend leukaemia integration 1 transcription factor	Paris Trousseau type thrombocytopenia/Jacobsen (11q23 del)	[8]
	*FYB*	FYN-binding protein	Novel thrombocytopenia	[26]
	*GATA1*	Erythroid transcription factor	GATA1 related disease (XLT and XLTT)	[9]
	*GFI1B*	Zinc finger protein Gfi-1b	Grey Platelet Syndrome + novel thrombocytopenia	[10]
	*HOXA11*	Homeobox protein Hox-A11	Amegakaryocytic thrombocytopenia with radio-ulnar synostosis	[74]
	*MPL*	Thrombopoietin receptor	Congentical amegakaryocytic thrombocytopenia	[12]
	*NBEAL2*	Neurobeachin-like protein 2	Grey Platelet Syndrome	[20]
	*RBM8A*	RNA-binding protein 8A	Thrombocytopenia with absent radii	[16]
	*RUNX1*	Runt-related transcription factor	Familial platelet disorder and predisposition to AML	[11]
	*THPO*	Thrombopoietin	Mild novel thrombocytopenia (heterozygous)	[13]
Platelet production	*ACTN1*	Alpha-actinin-1	Bleeding disorder, platelet-type 15	[36]
	*CYCS*	Cytochrome C	CYCS-related thrombocytopenia	[45]
	*GP1BA*	Platelet glycoprotein 1b alpha chain	Bernard–Soulier Syndrome + Platelet type von-Willebrand disease	[75]
	*GP1BB*	Platelet glycoprotein 1b beta chain		[76]
	*GP9*	Platelet glycoprotein IX		[77]
	*ITGA2B*	Integrin alpha-Iib	ITGA2B/ITGB3-related thrombocytopenia	[78]
	*ITGB3*	Integrin beta-3		[79]
	*MKL1*	MKL/myocardin-like protein 1	Thrombocytopenia with immunodeficiency	[31]
	*MYH9*	Myosin 9	MYH9 related disease	[80]
	*PRKACG*	cAMP-dependant protein kinase catalytic subunit gamma	Bleeding disorder, platelet-type 19	[39]
	*TUBB1*	Tubulin beta-1 chain	TUBB1-related macrothrombocytopenia	[29]
	*WAS*	Wiskott–Aldrich syndrome protein	Wiskott-Aldrich syndrome, X-linked thrombocytopenia	[81]
Platelet clearance/other	*ABCG5*	ABC transporter G family member 5	Thromobocytopenia associated with sitosterolaemia	[57]
	*ABCG8*	ABC transporter G family member 8		[57]
	*ADAMTS13* *GNE*	Disintegrin/metalloproteinase with thrombospondin motifs 13 Glucosamine (UDP-N-Acetyl)-2-Epimerase	TTP, Upshaw–Schulman syndrome GNE myopathy with congenital thrombocytopenia	[51] [65]
	*SLFN14*	Schalfen family member 14	Novel thrombocytopenia	[62]
	*STIM1* *vWF*	Stromal interaction molecule 1 Von Willebrand factor	Stormorken Syndrome Von Willebrand disease type 2B	[82] [83]

## Genes that effect megakaryocyte differentiation and maturation

Like all blood cells, megakaryocytes are derived from hematopoietic stem cells (HSCs) via progressively differentiated progenitor cells [2]. This is a process that is underpinned by the synthesis and synergistic effect of the cytokine thrombopoietin (TPO) through its receptor c-Mpl [3]. However, the commitment to differentiate and commit along the myelo-erythroid lineage is also highly regulated by transcription factors. One such transcription factor is GATA1, which is critically regulated transcriptionally [4]. Additional transcription factors such as Runt-related transcription factor 1, encoded by the gene *RUNX1*, ETV6 and FLI-1 in tandem with the transcriptional repressor GFI1B, ensure maturation of the megakaryocytes by binding critical promoter regions in crucial megakaryocyte-expressed genes [5, 6]. With the exception of Friend of Gata-1 (FOG1), the co-factor to GATA1, variants in the aforementioned hematopoietic transcription factors have previously been shown to cause inherited thrombocytopenia [7–11]. In addition, variants have also been observed in the TPO receptor gene *MPL*, as well as in its ligand, encoded by the gene *THPO* in one large Micronesian family with affected individuals displaying idiopathic aplastic anemia and mild thrombocytopenia [12, 13]. As the crucial role of TPO and transcription factors in megakaryocyte production is widely known, it is unsurprising that variants within these genes are commonly found within patients with inherited thrombocytopenia. However, clinical presentation does vary (most likely as a result of secondary effects of transcription factors and chemokines), but, all mutations are consistent in their ability to reduce proliferation of CD34+ cells by altering transcription profiles of genes crucial in megakaryocyte development [14].

In addition to the aforementioned genes involved specifically in the commitment to the proliferation towards megakaryocytes, a number of other genes have putative roles in differentiation and maturation. These include *HOXA11, RBM8A, ANKRD26* and *NBEAL2*.

Similar to FLI-1, ETV6 and AML-1, HOXA11 is a DNA-binding protein, which is expressed, like many other homeodomain box proteins, in human cord blood and hematopoietic precursor cells [15]. The specific molecular mechanism behind how variants in *HOXA11* cause amegakaryocytic thrombocytopenia with radio-ulnar synostosis syndrome has yet to be established. However, it has been suggested that a point mutation in the third helix of the homeobox domain can lead to a reduction in CD61 expression in staurosporine-induced K562 cells [15].

*RBM8A*, the gene encoding the exon junction splicing complex component Y14, is interesting both in the genetic complexity of the disease and in its proposed molecular mechanism. Thrombocytopenia with absent radii (TAR) syndrome is known currently as a result of the co-inheritance of a variably sized deletion of the region surrounding 1q21.1, including *RBM8A*, alongside one of two relatively high-frequency SNPs within the regulatory region of *RBM8A* [16]. The clinical outcome is severe but heterogeneous and seems to be determined by a number of factors, including variable gene expression and possible epigenetic modifiers. Little is known on the specific cellular effect of the compound hypomorphic effect of mutations within *RBM8A*, but a reduction in megakaryocyte progenitors is observed and is thought to arise as a result of aberrant JAK2 signaling downstream of TPO [17, 18].

The production of intracellular granules, during megakaryocyte maturation, from the budding of small vesicles containing cargo from trans-Golgi network is crucial for the further differentiation of platelets [19]. Platelet α-granules play a crucial role in platelet function and amplification of activation during hemostasis. A number of ITs, including *GATA1*-related thrombocytopenia (RT) and *GFI1B*-RT, show reduced numbers of platelet α-granules. However, a complete absence of platelet α-granules is observed in patients with Grey Platelet Syndrome (GPS), a condition caused by genetic variations within *NBEAL2* [20]. NBEAL2 localizes to the endoplasmic reticulum in platelets, where it is likely to function in membrane trafficking; however, the role of NBEAL2 in GPS and the subsequent reduction in platelet count is still largely unknown. *Nbeal2^−/-^* mice do recapitulate the phenotype of GPS, including an absence of platelet α-granules as well as delayed megakaryocyte maturation, decreased survival and decreased ploidy in cultured megakaryocytes [21]. Interestingly, however, a lack of *Nbeal2* in mice does not affect initial α-granule formation, packaging or transport to the budding platelets within megakaryocytes [22]. Both GPS mice and patients do develop premature myelofibrosis within the bone marrow, which may be as a result of spontaneous granule release or “leaking” from the megakaryocytes [22–24].

Another gene where variants have been shown to cause a defect in megakaryocyte maturation resulting in reduced platelet α-granules is *ANKRD26*. Like *RBM8A*, variants affect gene expression and occur in the 5’-UTR of the gene, specifically in a short stretch of nucleotides from c.-134G to c.-113A [25]. In affected individuals, megakaryocytes are small, with hypolobulated nuclei as a result of dysmegakaryopoiesis.

One of the most recently discovered genes known to cause inherited thrombocytopenia is *FYB*. Although the effect of the mutation has yet to be determined, a role in megakaryopoiesis has been suggested due to a reduced percentage of mature megakaryocytes in the bone marrow [26].

It seems then that the progression of HSCs through the megakaryocytic lineage to achieve mature megakaryocytes is a prime pathway to be disrupted by genetic mutation. Indeed, most variants not only involve altered expression or downstream signaling of the chemokine TPO, but many also affect additional signaling pathways, leading to a wide range of secondary abnormalities.

## Genes that affect pro-platelet formation and release

Following megakaryopoiesis, the next phase in platelet production is pro-platelet formation and release of platelets from pro-platelet tips [27]. As the expression profile alters to accommodate new functional processes of the megakaryocyte, so do the genes in which mutations mediate thrombocytopenia.

As megakaryocytes reach their mature state, they undergo fundamental changes to be able to release platelets into circulation via bone marrow sinusoids. These changes are underpinned by cytoskeletal rearrangements and subsequent cellular signaling. Causative mutations of IT are actually most commonly found to functionally disrupt these processes.

The fundamental action leading to the release of platelets is the production of proplatelet extensions from a demarcated membrane system. Once in the vascular niche, mature megakaryocytes produce protrusions by microtubule sliding [28]. β1-Tubulin is the main isoform within human megakaryocytes and mutations within the gene encoding *TUBB1* are known to cause an autosomal dominant form of inherited thrombocytopenia [29].

Like β1- tubulin, F-actin is present throughout proplatelets and allows proplatelet branching. These actin polymerization processes are promoted by nucleation factors such as WASp, encoded by *WAS*, which is selectively expressed in hematopoietic cells [30]. Mutations in *WAS* generally follow a genotype–phenotype correlation, where variants which abolish WASp expression or result in the expression of a truncated protein are associated with Wiskott–Aldrich Syndrome (WAS). Those causing decreased levels of WASp result in X-linked thrombocytopenia (XLT). Missense variants are most common in XLT and those occurring in the first three coding exons and the correlating EVH1/WH1 domain tend to cause the mild X-linked thrombocytopenia, which is not associated with the immunodeficiency observed in WAS.

Actin polymerization and the production of F-actin has recently been shown to be affected in a patient with a homozygous nonsense mutation within the gene encoding megakaryoblastic leukemia 1; *MKL1* [31]. In addition to severe immunodeficiency, the patient suffers from mild-to-moderate thrombocytopenia (platelet counts 50–150 × 10^9^/l). The cause of this is thought to be a loss of filamentous actin production due to a reduction in globular actin (G-actin) coupled with a reduction in megakaryocyte migration, which phenocopies mice deficient for murine Mkl1 [32, 33].

Myosins, in particular, IIA and IIB, are also involved in proplatelet formation by interacting with actin filaments generating contractile forces. One myosin, the heavy chain of nonmuscle myosin IIA (NMMHC-IIA) encoded by the gene *MYH9*, is thought to play a slightly different role as mutations actually increase proplatelet formation [34]. Mutations causing *MYH9*-related disease therefore cause thrombocytopenia through the most proximal part of platelet formation, where a disruption in the Rho-Rho kinase-myosin IIA pathway leads to a lack of reactivation of NMMHC-IIA. This is a process that is required for the budding of preplatelets and subsequent separation into platelets; therefore, mutations in *MYH9* cause a loss of induced fragmentation of preplatelets promoting the formation of a reduced number of large platelets [35].

Cross linking of actin filaments is required for their binding to the actin cytoskeleton. This is mediated in structures known as actin-binding domains (ABDs), which contain α-actinin. One of the four isoforms, which is expressed in megakaryocytes, is ACTN1. To date, 13 pathogenic missense variants have been found within the encoding gene *ACTN1;* all cause a mild dominantly inherited thrombocytopenia where megakaryocyte tips are reduced in number, increased in size and exogenous expression of the mutations inhibits actin filament assembly [36].

Filamins, via an N-terminal ABD, anchor the filaments to the cell membrane. Variations in the most abundant filamin in platelets, *FLNA*, are known to cause periventricular nodular heteropia as well as an isolated thrombocytopenia [37]. Some patient megakaryocytes from cultured peripheral CD34+ blood cells show frayed structures and signs of cytoplasmic degradation favoring abnormal distribution into incorrectly packaged platelet-like fragments. However, a population of platelets negative for FLNA was observed, indicating it may not be necessary in proplatelet formation, therefore, playing a noncrucial role [37].

There may remain an important role for FLNA in platelet biogenesis as its proteolysis is protected by phosphorylation at S2152 by cAMP-dependent protein kinase A, which is similar to the protection observed in GP1bβ [38]. The kinase consists of two catalytic subunits, the y-isoform of which is encoded by *PRKACG*. To date, only one variant has been found within *PRKACG*, which results in rapid degradation of FLNA and a reduction in the percentage of proplatelet-bearing megakaryocytes [39].

Complementary to the physical formation of proplatelets is the relay of extracellular signals to the cytoskeleton via membrane-bound receptors. Two main receptors are affected by mutations in inherited thrombocytopenia. The first is the receptor for Von-Willebrand factor (VWF), GP1b-IX-V. Consisting of four subunits, GP1bα, GP1bβ, GPIX and GPV, activation of the receptor via interaction of VWF with GP1bα relays signals to aid in proplatelet formation. Variants in the encoding genes, *GP1BA, GP1BB and GP9*, are known to cause both monoallelic and biallelic forms of BSS and lead to a reduction in proplatelet-forming megakaryocytes in culture [40, 41].

The second receptor known to be affected in the spectrum of inherited thrombocytopenias is the integrin GPIIb-IIIa, the receptor for fibrinogen. Encoded by the genes *ITGA2B* and *ITGB3*, mutations are associated with Glanzmann thrombasthenia (GT). GT often causes bleeding with no alteration in platelet count; however, gain of function variants exists that lead to a thrombocytopenia, suggesting a role for the interaction of fibrinogen for platelet biogenesis [42]. Recently, it has been suggested that cytoskeleton rearrangement can be impaired, arresting actin turnover at the stage of polymerization, by permanent triggering of the aIIbβ3-mediated outside-in signaling [43]. Murine megakaryocytes transduced with an *ITGB3* variant (c.del647-683) generated proplatelets with a reduced number of large tips and barbell-proplatelets, suggesting impaired cytoskeletal remodeling as the cause of thrombocytopenia in patients with GT.

One gene whose involvement in platelet production currently, through the intrinsic apoptosis pathway, is controversial is Cytochrome C (*CYCS*). CYCS initiates apoptosis through the activation of caspase 9 and subsequently caspase 3, a dysregulation of which was originally suggested as a feature of platelet production [44, 45]. To date, two variants (Y49H [p.48 in original publication] and G42S [p.41 in original publication]) have been observed in the apoptosis-linked gene *CYCS* [45, 46]. Indeed, electron microscopy of patient bone marrow shows intramedullary naked megakaryocyte nuclei, indicative of dysregulated megakaryopoiesis and premature platelet release. Furthermore, mouse lung fibroblasts transduced with *Cycs^Y48H^* and *Cycs^G41S^* show increased apoptotic activity in response to staurosporine. It was therefore first believed that apoptosis had a fundamental role in the timing of pro-platelet release, hypothesizing that inhibition of actin polymerization, a process required for the formation of proplatelets, activates the apoptotic pathway [47]. However, platelet production can proceed regardless of both the intrinsic and extrinsic apoptotic pathways, suggesting there is more to the role of apoptosis in megakaryopoiesis than currently meets the eye [48].

## Genes with a molecular function outside of platelet production

**Figure 1.**
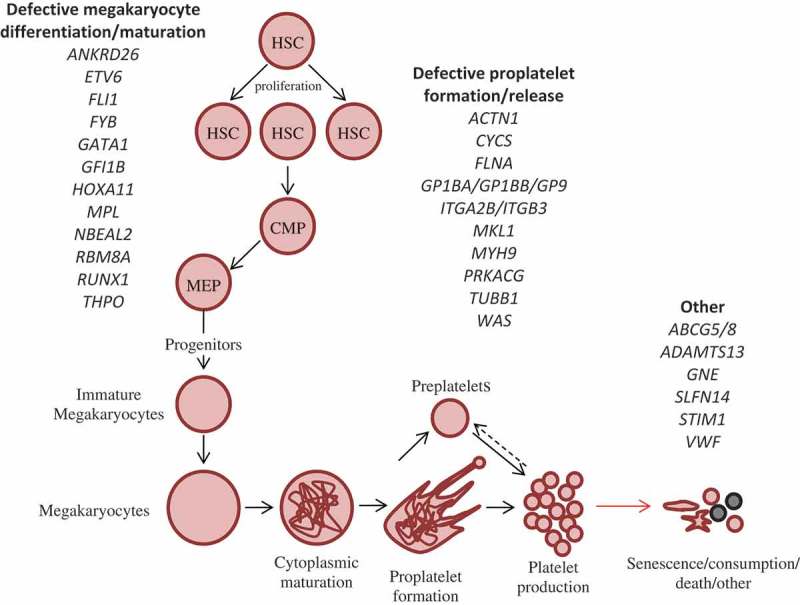
Megakaryopoiesis, platelet production and other causes of IT. Differentiation from HSCs to platelets proceeds by a number of intermediate cell types, which leads to the formation of megakaryocytes which fragment via proplatelet formation to produce mature platelets. This process is driven by a number of genes encoded a number of transcription factors and proteins. Defects in these genes have been shown to give rise to thrombocytopenia by mostly affecting the relevant stage of platelet production they are labeled under. Variants that do not play a role in platelet production by megakaryopoiesis are included in the third subgroup entitled platelet removal, death and other. HSC: Hematopoietic stem cell, CMP: Common myeloid progenitor, MEP: Megakaryocyte-erythroid progenitor.

It is clear and obvious that the fundamental processes governing the production of platelets are easily affected by deleterious mutations leading to thrombocytopenia. This is summarized in Figure 1. However, disease affecting megakaryocytes and the formation of platelets do not cover all of the genes currently known to cause thrombocytopenia. These alternative genes do not play role in megakaryopoiesis/thrombopoiesis. Their molecular causes of disease are unique or not currently known and it is best to consider them individually and separate from the aforementioned genes.

VWF is particularly well known due to its importance in hemostasis, illustrated with the functional deficiency of vWF known as von Willebrand disease (VWD), a well-characterized and common collection of bleeding disorders. One particular type of VWD is known as type 2B, which refers to gain-of-function mutations that increase the affinity of vWF for GP1b on the platelet surface. The net effect of this is the presence of giant platelets due to continuous interactions between vWF and GP1bα disrupting megakaryopoiesis [49]. More interestingly, though, large multimers of vWF associated with platelets are also observed in VWD type 2B patients [50]. In vWF knock-in mice, these specific complexes are taken up efficiently by macrophages in the liver and spleen, indicating an increased clearance of platelets from the circulation.

One interesting gene with a mechanism of pathogenesis for thrombocytopenia is *ADAMTS13*. Variants in the metalloproteinase *ADAMTS13* are the genetic cause of Upshaw–Schulman syndrome, alternatively known as congenital thrombotic thrombocytopenic purpura (TTP) [51]. TTP is a life-threatening systemic illness characterized by hemolytic anemia, neurological symptoms, renal dysfunction as well as thrombocytopenia. Normally in-circulation ADAMTS13 is the von Willebrand factor-cleaving protease. However, loss-of-function mutations within *ADAMTS13* and subsequent TTP result in vWF not being cleaved at the Tyr 842-Met 843 peptide bond resulting in the accumulation of large vWF multimers similar to a gain-of-function mutations in *vWF*[52]. Unlike VWD type 2B, the reduction in platelet count observed in TTP may be due to platelet aggregation under sheer stress because of an inability to cleave vWF [53]. This has the propensity to explain vWF–platelet aggregation in the arterioles and capillaries, a phenotype characteristic of TTP.

An increased clearance of platelets is also present in patients with activating mutations of *STIM1* within Stormorken syndrome and more recently York syndrome [54, 55]. Platelets in these patients are circulating in a preactivated state due to a constitutively active store operated Ca(2+) release-activated Ca(2+) (CRAC) channel and increase in Ca2+ entry. The net effect of which causes a reduction in the number of circulating platelets, but not due to an early ageing of platelets but an increased clearance of pre-activated platelets within the spleen, which is recapitulated in *Stim1Sax^/+^* mice [56].

One interesting case of thrombocytopenia is as a secondary symptom with Sitosterolemia. Sitosterolemia is a rare, autosomal recessive disease caused by mutations in two adjacent ATP-binding cassette transport genes; *ABCG5* and *ABCG8* [57]. Hematological abnormalities, including thrombocytopenia, are a symptom of this complex disorder which results in decreased excretion and therefore accumulation of dietary sterols. *ABCG5* and *ABCG8*, which normally act as a heterodimeric efflux pump, are not present on blood cells or platelets. The effects of mutations are therefore considered to be because of the relative toxicity they possess in high levels to cellular membranes [58, 59]. Interestingly, in ABCG5/ABCG8 knock-out mice, which are fed on high-plant-sterol diet, platelets are noted to be hyper-activatable and show impaired function [60].

One of the most recently discovered genes known to cause inherited thrombocytopenia is *SLFN14*. SLFN14 has recently been identified as an endoribonuclease, functioning to destroy mRNA, which cannot be correctly translated, and causes degradation of ribosomal subunits [61]. Three consecutive heterozygous missense mutations were identified in three unrelated families predicted to encode substitutions K218E, K219N and V220D [62]. Alongside the reduced number of platelets, they also show platelet dysfunction and an increased immature platelet fraction, a noninvasive measure indicative of increased platelet clearance. However, the exact mechanism through which mutations in *SLFN14* mediate thrombocytopenia and aberrant platelet function is unknown.

There is similar lack of knowledge for the molecular effect of one of the most recently reported causative genes of IT, *GNE*, Mutations within the gene encoding Glucosamine (UDP-N-Acetyl)-2-Epimerase/N-Acetylmannosamine kinase (GNE) are most noted as the molecular cause of Sialuria (OMIM#269921) and Hereditary Inclusion Body Myopathy (HIBM; OMIM#600737) [63, 64]. Recently, however, two related individuals presented with GNE myopathy associated with congenital thrombocytopenia [65]. Both patients suffered from a severe reduction in platelet count; however, the hematological effect of mutations within *GNE* has yet to be suggested.

Clearly, it is not solely platelet production that is affected by deleterious mutation. Lifespan can be inadvertently altered by pre-activation, the production of aggregated multimers and by toxicity. However, with platelet lifespan so intrinsically controlled by levels of pro-apoptotic proteins versus pro-survival proteins, why is it that no variants have thus far been discovered in this specific pathway? For us to understand this further, we need to consider how platelet lifespan is controlled and how it could theoretically be disturbed.

In platelets, it was long suggested that the Bcl-2 family of pro-survival proteins was required to suppress the pro-apoptotic signals of BAK and BAX [66]. It was Mason *et al*. who first established the link of degradation of Bcl-X_L_ (encoded in humans by *BCL2L1*) to platelet death [67]. This subsequent loss of the restrained pro-apoptotic signal was therefore suggested as a molecular clock for platelet lifespan, an idea which was pioneered by the group of B. Kile. [67]. Megakaryocyte-specific deletion of *Bcl-x_L_* in mice does indeed profoundly reduce the platelet count to 2% of that observed in wild-type mice [68]. Additionally, platelet survival is markedly increased in *Bak and Bax* knock out and megakaryocyte-specific depleted mice [68].

So in a process so highly controlled by expression and protein levels, why are there not any variants in Bcl-x_L_ or Bak/Bax that are known to cause thrombocytopenia?

There are two possible explanations. One is that loss-of-function variants in *BCL2L1* are lethal. While Bcl-X_L_ is involved in preventing mature megakaryocytes from apoptosis it is not essential alone for their growth and development, whereas presence of either Bcl-X_L_ or Mcl-1, another member of the pro survival Bcl-2 family, is required for organism viability [69]. However, Bcl-X_L_’s functions are not limited solely to platelet lifespan and it follows a wide expression pattern both in adult and in embryonic tissue [70, 71]. Interestingly, Jak2, which is thought to positively regulate Bcl-X_L_ expression in megakaryoblastic cells, is associated with decreased survival when selectively deleted in mouse hematopoietic cells [69, 72]. Therefore, at this moment in time, it is difficult to rule out the possibility that an organism-specific effect may be occurring within humans where genetic variations in *BCL2L1* are simply not viable.

The other point to consider is the possibility that we haven’t determined a molecular cause of disease within *BCL2L1* or any other genes involved in platelet lifespan simply due to their rarity. The most recent large-scale targeted genomics study into inherited thrombocytopenia revealed that only 50% of patients have a defined genetic etiology of disease [73]. With the progression of next-generation sequencing and the introduction of large-throughput whole exome and whole genome sequencing, new variants are being discovered in these previously undiagnosed patients [7, 62]. Inherited thrombocytopenias though, as a spectrum of disorders, are still fundamentally underdiagnosed. Yet, as our knowledge increases, we learn more about the effect of deleterious variants gaining insights into new mechanisms within platelet production, function and lifespan.
